# Molecular and Cellular Mechanisms of Melatonin in Osteosarcoma

**DOI:** 10.3390/cells8121618

**Published:** 2019-12-12

**Authors:** Ko-Hsiu Lu, Renn-Chia Lin, Jia-Sin Yang, Wei-En Yang, Russel J. Reiter, Shun-Fa Yang

**Affiliations:** 1 Department of Orthopedics, Chung Shan Medical University Hospital, Taichung 402, Taiwan; cshy307@csh.org.tw (K.-H.L.); cshy594@csh.org.tw (R.-C.L.); 2 School of Medicine, Chung Shan Medical University, Taichung 402, Taiwan; 3 Division of Hyperbaric Oxygen Therapy and Wound Medicine, Chung Shan Medical University Hospital, Taichung 402, Taiwan; 4 Institute of Medicine, Chung Shan Medical University, Taichung 402, Taiwan; gazn_sheep@yahoo.com.tw (J.-S.Y.); weienyang@gmail.com (W.-E.Y.); 5 Department of Medical Research, Chung Shan Medical University Hospital, Taichung 402, Taiwan; 6 Department of Cell Systems and Anatomy, UT Health, San Antonio, TX 78229, USA

**Keywords:** apoptosis, melatonin, metastasis, osteosarcoma, pathway

## Abstract

Osteosarcoma, the most common primary bone malignancy, occurs most frequently in adolescents with a peak of incidence at 11–15 years. Melatonin, an indole amine hormone, shows a wide range of anticancer activities. The decrease in melatonin levels simultaneously concurs with the increase in bone growth and the peak age distribution of osteosarcoma during puberty, so melatonin has been utilized as an adjunct to chemotherapy to improve the quality of life and clinical outcomes. While a large amount of research has been conducted to understand the complex pleiotropic functions and the molecular and cellular actions elicited by melatonin in various types of cancers, a few review reports have focused on osteosarcoma. Herein, we summarized the anti-osteosarcoma effects of melatonin and its underlying molecular mechanisms to illustrate the known significance of melatonin in osteosarcoma and to address cellular signaling pathways of melatonin in vitro and in animal models. Even in the same kind of osteosarcoma, melatonin has been sparingly investigated to counteract tumor growth, apoptosis, and metastasis through different mechanisms, depending on different cell lines. We highlighted the underlying mechanism of anti-osteosarcoma properties evoked by melatonin, including antioxidant activity, anti-proliferation, induction of apoptosis, and the inhibition of invasion and metastasis. Moreover, we discussed the drug synergy effects of the role of melatonin involved and the method to fortify the anti-cancer effects on osteosarcoma. As a potential therapeutic agent, melatonin is safe for children and adolescents and is a promising candidate for an adjuvant by reinforcing the therapeutic effects and abolishing the unwanted consequences of chemotherapies.

## 1. Introduction

Cancer is a well-known public health problem associated with high mortality and disability rates worldwide [1]. According to the data reported by the World Health Organization (WHO), new cancer-related cases have increased to 18.1 million and cancer-induced deaths to 9.6 million in 2018 [2]. Cancer is a leading cause of death for children, with 300,000 new cases diagnosed each year. Osteosarcoma is the most prevalent primary bone cancer with a peak of incidence at 10–15 years and the second incidence peak in older adulthood [3,4,5]. This malignant bone tumor often first develops in the metaphysis of long bones (distal femur and proximal tibia) in about six in every million children and two in every million adults [6]. It is therefore feasible that there may be a relationship between the incidence of osteosarcoma and the rate of bone growth [4].

Even though surgical en bloc resection of the cancer or amputation of the extensive diseased extremity to achieve a complete radical excision has been the treatment of choice for osteosarcoma, the main cause of most treatment failures and the high mortality rate is still its highly metastatic potential [7]. Reassuringly, the combination of surgery and chemotherapy for osteosarcoma has increased the long-term survival chances of approximately 68% through limb-sparing surgeries based on radiological staging, surgical techniques, and new chemotherapy protocols [8]. Nonetheless, potent metastatic transfers to the lungs are still responsible for most treatment failures and are accountable for one of the most lethal pediatric malignancies. Thus, novel agents that target particular intracellular pathways related to the distinctive properties of osteosarcoma cells need to be developed.

Melatonin levels, which are high in children, with the highest peak between ages four–seven [9], begin to decrease prior to the onset of puberty and continue to decline during puberty [10]. Initially, it is rather bewildering how such findings, compared with the incidence of osteosarcoma, have generally received little attention. After establishing a hypothesis of a correlation between the declination in melatonin levels (with provides oncostatic protection) and the increase in the chances of developing the fatal osteosarcoma in young patients, melatonin has been studied for its anti-osteosarcoma action and as an adjunct to the conventional chemotherapy for osteosarcoma to improve the prognosis of the fatal disease [11]. Moreover, melatonin is non-toxic and can augment anticancer action while ameliorating the side effects of many other chemotherapeutic drugs. Therefore, it is not surprising that increasing attention has been devoted to melatonin as it may be integrated into adjuvant therapies to amplify the therapeutic effects and to diminish the side effects of chemotherapies [12].

## 2. Biosynthesis, Metabolism, Actions, and Signaling Pathways of Melatonin

### 2.1. Biosynthesis of Melatonin

**Figure 1:**
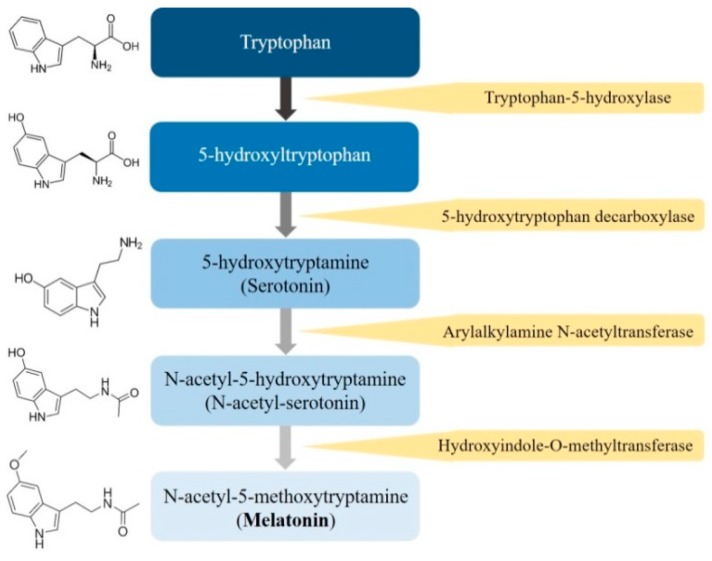
The process of the melatonin’s biosynthesis.

Melatonin (*N*-acetyl-5-methoxytryptamine), a ubiquitously amphiphilic indole amine, is synthesized from its precursor, tryptophan, and is secreted primarily by the pineal gland and several other organs, including the retina, gastrointestinal tract, bile, skin, bone marrow, and lymphocytes, of humans and mammals [13,14]. In mammals, melatonin synthesis is dependent on the day-night cycle and is part of an intricate enzymatic pathway in which tryptophan is hydroxylated to 5-hydroxytryptophan by tryptophan-5-hydroxylase [15] (Figure 1). Then, 5-hydroxytryptophan is decarboxylated into serotonin by 5-hydroxy-l-tryptophan decarboxylase; afterwards, serotonin is acetylated into *N*-acetylserotonin, and *N*-acetylserotonin is subsequently methylated to melatonin, which is quickly released into the bloodstream.

### 2.2. Metabolism of Melatonin

As a circadian pattern, the synthesis and secretion of melatonin is regulated by the central circadian clock, which is located in the suprachiasmatic nucleus of the hypothalamus [16], and its synthesis is stimulated by the night and inhibited by light [17]. As in instances of reduced light exposure, norepinephrine is released from sympathetic nerve endings to strengthen the intracellular levels of c-AMP and activates the protein kinase A, which in term regulates the function of arylalkylamine N-acetyltransferase to limit the rates of melatonin production [15]. The levels of melatonin in cerebrospinal fluid and blood reach maximal concentration near the middle of the darkest period (between 02:00 and 04:00) and decrease throughout the daytime [18]. Nearly 80% of the melatonin is synthesized at night with nocturnal serum concentrations varying between 80 and 120 pg/mL and low serum concentrations (10–20 pg/mL) during daylight hours [9]. After an oral intake of 500 μg melatonin, the mean (± SD) half-life of the elimination is 47 ± 3 min [19]. The clearance of melatonin is rapid through the liver by cytochrome P450 enzymes CYP1A1 and 1A2 enzymes undergoing 6-hydroxylation to 6-hydroxymelatonin [20]. Large amounts of the metabolites are secreted into the bile [21] with minor amounts being further conjugated with sulfuric acid (90%) or glucuronic acid (10%) and being excreted in the urine as 6-sulfatoxymelatonin [22]. Additionally, about 5% of serum melatonin is excreted as the unmetabolized drug [23]. In case of intravenous administration, the hepatic bio-degradation is less important due to the lack of hepatic first pass.

### 2.3. Actions of Melatonin

While melatonin is regulated by the central circadian clock, it transmits during the nighttime and helps organize target organs and organ systems into appropriate homeostatic metabolic rhythms [24] and also reverses to modulate the central circadian clock and peripheral oscillators in tissues and organs, which makes melatonin a circadian pacemaker [25]. Meanwhile, circadian secretion of melatonin also synchronizes the immune system via a reciprocal association. As the relevance of melatonin upholds the circadian rhythm and orchestrates many signaling pathways, melatonin is not only a hormone, but also a cell protector [26] involved in immunomodulation, anti-oxidative processes, and hematopoiesis [20]. Consequently, a disruption of the circadian rhythm can interfere with the nocturnal melatonin signal to disturb normal homeostatic metabolic rhythms and many physiologic cell functions, which, if deregulated, may cause an accelerated pace towards cancer [27]. In addition to sleep induction, biological rhythms resynchronization, anti-oxidation, and anti-inflammation, melatonin possesses a wide spectrum of biological effects such as immunomodulation and apoptosis induction [28]. Furthermore, melatonin is involved in the regulation of immune functions and the tumor microenvironment and acts as an anticancer agent. Hence, it is not surprising that melatonin has been popularly used as a healthcare product in the global market.

### 2.4. Signaling Pathways of Melatonin

The amphiphilic indole amine is capable of easily penetrating into cells and exerting various biological effects that either attributes to its interactions with the cell surface and intracellular receptors or directs effects as a radical scavenger [29]. By affecting its receptors or acting as a direct antioxidant agent, melatonin modulates many signaling pathways essential for cellular functions [26], such as cellular metabolism, DNA damage response, cell-to-cell communication, and more. As a result, melatonin behaves via receptor-dependent and receptor-independent mechanisms for its important oncostatic properties [30]. The receptors affected by melatonin belong to the G protein coupled receptors superfamily, including three subtypes of MT1, MT2, and MT3. In mammals, MT1 (encoded by *MTNR1A*) and MT2 (encoded by *MTNR1B*) are mainly responsible for regulating the melatonin’s downstream effects [31].

The MT1 receptor, mainly located in the pars-tuberalis of the pituitary gland, the suprachiasmatic nuclei of the hypothalamus [16], the skin, and the retina, is believed to be involved in the inhibitory effect of melatonin in mammalian brains to act as a master regulator of brain function. The MT2 receptor, which is widely distributed in osteoblasts, vessels of extremities, and the retina, is active in the phase shifting of the circadian clock [32]. However, the receptor-independent mechanisms are related to the prevention of circadian disruption, antioxidant activity, tumor metabolism and cancer immunity, regulation of apoptosis, and inhibition on angiogenesis and migration [30,33]. Melatonin’s anticancer properties in many cancers have recently attracted attention; thereby, the role of melatonin in the treatment of cancer cells has been extensively studied and the number of articles has been increasing rapidly, given that amplification of the anticancer properties constitutes a possible treatment feature in osteosarcoma. Recently, a compelling body of evidence has documented significant anti-osteosarcoma effects of melatonin in vitro and in vivo.

## 3. Function of Melatonin Relating to the Bone

### 3.1. Effects of Melatonin on Mesenchymal Stem Cells

**Figure 2:**
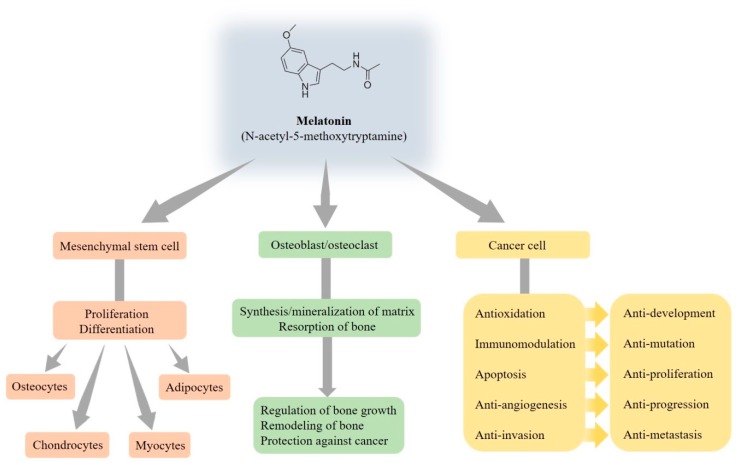
Multiple functions of melatonin relating to the bone.

It is widely demonstrated that melatonin is able to regulate signaling pathways that drive commitment and the differentiation of mesenchymal stem cells, which are multipotent progenitor cells, into several cell types such as osteocytes, chondrocytes, myocytes, and adipocytes [34] (Figure 2). Mesenchymal stem cells’ differentiation is finely regulated by the action of mechanical and molecular signals from the extracellular environment, but melatonin may also be an important regulator of precursor cell commitment and differentiation [35]. It is universally approved that adipogenesis and osteogenesis is a reciprocal relationship in bone marrow. Despite no apparent effect on the proliferation of human mesenchymal stem cells, melatonin directly inhibits adipogenic differentiation toward the adipocyte lineage and simultaneously fosters osteogenic differentiation by suppressing peroxisome proliferator-activated receptor γ (PPARγ) expression and enhancing Runt-related transcription factor 2 (Runx2) expression [36]. Furthermore, melatonin enhances the differentiation of human mesenchymal stem cells to osteoblasts via MT2 receptors and the mitogen/extracellular signal-regulated kinase (MEK)/extracellular signal-regulated kinase (ERK) 1/2 signaling cascade [37].

### 3.2. Effects of Melatonin on Osteoblasts and Osteoclasts

In addition to the regulation of the sleep cycle, endocrine system, reproductive cycle, bone metabolism, cell cycle, and mitochondrial function, melatonin influences bone growth and metabolism to promote the differentiation and mineralization of osteoblast cells grown [38]. Additionally, melatonin participates in the regulation of bone homeostasis via the modulation of osteoblast and osteoclast activities by receptor-independent and dependent pathways, and then bone remodeling is established by the synthesis of bone matrix by osteoblasts and the resorption of bone by osteoclasts [39]. Meanwhile, melatonin induces osteoblastic differentiation and mineralization for osteogenesis and bone formation through the bone morphogenetic protein (BMP)/ERK/Wnt signaling pathways [40].

Disrupted circadian rhythms alter the expression of clock genes and deregulate oncogenes, which ultimately promote tumor development and progression. Melatonin indeed represses in vitro osteoblast differentiation and the mineralization of the matrix to regulate bone growth [41,42], so the fall of melatonin has been shown to be an indicator of skeletal maturation [43]. Recent studies have reported that melatonin possesses multiple tumor-suppressing properties for a myriad of tumors [30,33,44,45]. It is worthy to mention that individuals with distinct defects in osteoblast functions are at an increased risk of contracting cancer, indicating that osteosarcoma may be partly related to abnormal osteoblast functions. However, melatonin, on the contrary, possesses the ability to enhance normal osteoblast functions and thus plays a protective role against cancer.

### 3.3. Effects of Melatonin on Cancer Cells

Melatonin has been sparingly investigated to counteract tumor growth and metastasis through various mechanisms, depending not only on different cancer types, but also the different cell lines in the same kind cancer. Even the actions of melatonin between cancer cells and normal cells are obviously different [30,44,45,46,47,48]. For example, melatonin is antiapoptotic in normal cells [49], but proapoptotic in cancer cells [50]. Due to the widespread subcellular distribution and free radical scavenging actions, numerous studies have suggested that melatonin abrogates oxidative damage to inhibit cancer development [51], and this relates, in part, to its pro-oxidative actions in cancer cells [50]. To enhance the immunomodulatory potential of the destined cell mutation, melatonin augments the immune responses and alleviates immunodeficiency states [52], along with enhancing immunosurveillance by stimulating the activities of immune cells, including T and B lymphocytes, monocytes, macrophages, and natural killer cells, and stimulating the several cytokine productions, for example, interferon-γ, interleukin (IL)-1, IL-2, IL-6, and tumor necrosis factor-α [53]. As a result of accumulating evidence, melatonin possesses pleiotropic bioactivities and exhibits a very wide antitumor repertoire.

As mentioned earlier, the role of melatonin in inhibiting tumor proliferation has been well documented [48]. Melatonin has directed proapoptotic on cancer cells, limits the cellular uptake of key factors for tumor growth and their signaling molecules [54], and inhibits cell cycle kinetics and telomerase activity to restrain tumor cell growth [55]. To suppress cancer progression and metastasis, melatonin reduces vascular endothelial growth factor secretion and the formation and release of endothelin-1 to inhibit angiogenesis [56]. Through the remodeling of the extracellular matrix (ECM), the reorganization of the cytoskeleton, and epithelial–mesenchymal transition (EMT) [47,48], melatonin counteracts the angiogenic responses and thus abrogates cancer cell invasion and metastasis.

## 4. Molecular Actions of Melatonin in Osteosarcoma

### 4.1. Cytotoxic Activity of Melatonin in Human Osteosarcoma

Apoptosis, or programmed cell death, a key regulator of physiological growth control and regulation of tissue homeostasis, is characterized by typical morphological and biochemical hallmarks, including cell shrinkage, nuclear DNA fragmentation, and membrane blebbing [57]. To undergo apoptosis, the activation of an important initiator and effector caspases would be initiated through the activation of the extrinsic (receptor) pathway or the stimulation of the intrinsic (mitochondria) pathway [58,59]. Multiple stress-inducible molecules, such as mitogen-activated protein kinase (MAPK)/ERK, c-Jun N-terminal kinase (JNK), and nuclear factor-κB, have been implied in transmitting the apoptotic pathway [60,61]. Currently, most anticancer strategies in clinical oncology focus on triggering apoptosis in cancer cells. On the contrary, failure to undergo apoptosis may result in treatment resistance. Thereby, understanding the molecular events that regulate apoptosis in response to chemotherapy provides novel opportunities to develop the molecular targeted therapy through the intrinsic and/or extrinsic pathways for the intractable osteosarcoma.

**Figure 3:**
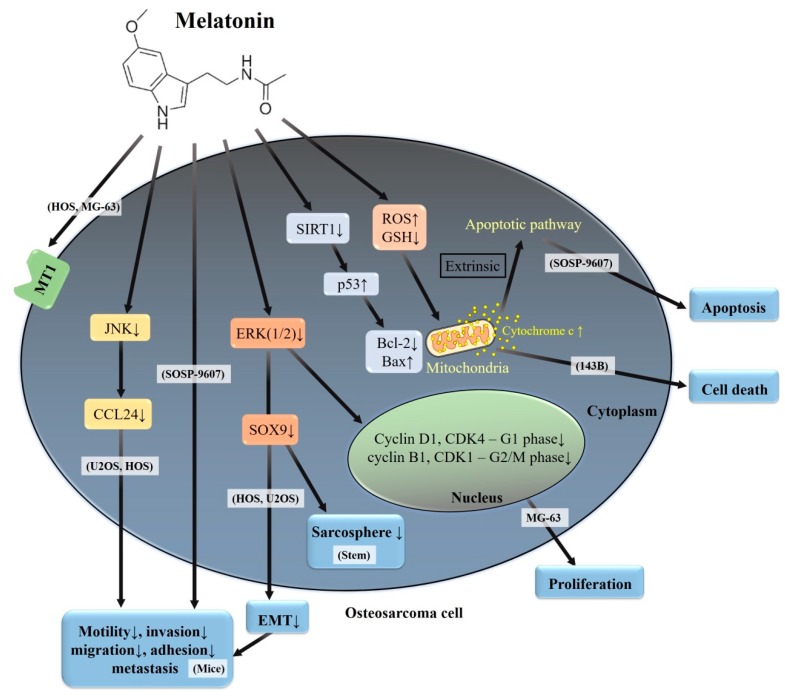
A summary of various signaling pathways involved in melatonin on human osteosarcoma. MT1: melatonin receptor 1; ERK: extracellular signal-regulated protein kinase; JNK: c-Jun N-terminal kinase; ROS: reactive oxygen species; GSH: glutathione; Bcl-2: B-cell lymphoma/leukemia 2; BAX: Bcl-2-associated X protein; SIRT1: sirtuin 1; EMT: epithelial–mesenchymal transition; CCL24: C-C motif chemokine ligand 24; and MG-63, 143B, HOS, U2OS, and SOSP-9607: human osteosarcoma cell lines; Stem: osteosarcoma stem cells.

Compelling evidence has highlighted that melatonin demonstrates both cytotoxic and anti-metastatic activities in various cancer cells and they appear to be cell-type specific—even in human osteosarcoma cell lines (Table 1). Abundant expression of MT1-mRNA in human osteosarcoma HOS and MG-63 cells and other malignant and non-malignant bone tumors has been demonstrated [62], and these findings suggest an irreplaceable role for MT1 in bone pathology (Figure 3). In human osteosarcoma 143B cells, 100 μM of melatonin reduces mitochondrial reactive oxygen species (ROS) generation, cell death, and mitochondrial ROS-induced depletion of cardiolipin in order to improve the retardation of mitochondrial movement and dynamics [63]; however, lower concentrations (10^−5^–10^−13^ M) of melatonin have no effect on the growth, morphology or cell cycle [64]. It seems that the adequate concentration of melatonin exhibits a considerable potential for rescuing cardiolipin-dependent mitochondrial dynamics-associated mitochondrial pathologies in 143B cells.

Through the downregulation of the expressions of cyclin D1 and CDK4 (related to G1 phase), as well as cyclin B1 and CDK1 (related to G2/M phase), melatonin inhibits the proliferation of human osteosarcoma MG-63 cells [65]; meanwhile, the inhibition of the ERK1/2 signaling pathway is involved in the melatonin’s anti-proliferative effect, induction of G1 and G2/M phase cell arrest, and downregulation of expressions of cyclin D1, CDK4, cyclin B1, and CDK1 in MG-63 cells [66]. Furthermore, melatonin affects the cellular redox status to create a substantial correlation between apoptosis and ROS generation in human osteosarcoma SOSP-9607 cells [67]. In addition to the alleviation of the adhesive and migratory abilities, melatonin induces apoptosis in SOSP-9607 cells via the down-regulation of sirtuin 1 (SIRT1, a conserved nicotinamide adenine dinucleotide-dependent deacetylase), which has an association with age and cancer [68], thus leading to increased p53 acetylation. Then, acetylated p53 triggers the intrinsic apoptotic pathway by decreasing Bcl-2 levels and increasing Bax and cytochrome c expressions.

### 4.2. Anti-Metastatic Effects of Melatonin in Human Osteosarcoma

As the tumor develops, it becomes increasingly important for the cancer cells to sustain their growth, to increase the tumor size through angiogenesis process, and to metastasize throughout the body. Rampant metastasis of cancerous cells is a serious feature of all malignancies and most cancer morbidities and mortalities are associated with metastatic spread. Accordingly, it is a significant goal to bring insight in order to understand the signaling pathways underlying the complicated process of metastasis that occurs through a series of steps termed the invasion–metastasis cascade [69]. Briefly, the invasion–metastatic cascade pathways include the detachment of cancer cells and degradation of ECM, induction of cellular motility, invasion and migration, adhesion themselves to endothelial cells, and re-establishment of cancer cell growth at a distant site [70].

It is generally accepted that EMT in the developmental program plays a critical role in promoting metastasis in epithelium-derived carcinoma throughout the metastasis process. In contrast, sarcoma cells of mesenchymal origin embed themselves inside the ECM and rarely establish tight contact with neighboring cells. During the past decade, the mechanism of EMT involvement in invasion and migration allows carcinoma cells to dissociate from each other and to degrade ECM, but the investigators further applied the mechanism to sarcoma cells, e.g., osteosarcoma, with the ability to initiate the invasion–metastasis cascade [47,48,71,72,73]. In this regard, sarcoma cells make ECM easier and it may be one of the reasons why osteosarcoma, arising from the aberrant transformation of mesenchymal cells, exhibits a highly metastatic potential. Regrettably, lung metastasis is the main cause of death rather than the primary tumor itself [74].

Of the sirtuin family members, SIRT1 is the most well-known to participate in a number of processes, including cell growth, variability, p53 acetylation, DNA damage, and apoptosis [75,76]. As mentioned before, higher concentrations (up to 1000 μM) of melatonin induce apoptosis in human osteosarcoma SOSP-9607 cells via the down-regulation of sirtuin 1, leading to p53 hyperacetylation, whereas lower concentrations (12.5–50 μM) of melatonin inhibit SOSP-9607 cellular adhesion and migration [67] (Table 2). Another in vitro study shows that melatonin potently suppresses migration and invasion in human osteosarcoma HOS and U2OS cells, and inhibits the sarcosphere formation of osteosarcoma stem cells via the down-regulation of SOX9-mediated signaling pathway [77]. Additionally, melatonin suppresses EMT via the downregulation of SOX9-mediated signaling to inhibit migration and invasion of HOS and U2 cells and decreases tumor initiating cells and lung metastasis of osteosarcoma in the nude mice model. Likewise, melatonin significantly downregulates the expression of mesenchymal marker vimentin, as well as decreases the expression of β-catenin and N-cadherin, indicating that melatonin can partially block the EMT progress of U2 cells. Furthermore, p-ERK is prominently downregulated and the transcription factor SOX9 expression level is significantly repressed under the melatonin treatment, indicating that melatonin might suppress the EMT program and stem cells in U2 cells through the ERK pathway and downregulation of SOX9 expression. Collectively, in vitro and in vivo evidence provides that SOX9 has a crucial role in mediating melatonin-dependent inhibition of the initiation and metastasis of HOS and U2 cells.

While melatonin, up to 2.0 mM, has no cytotoxic effects, it intriguingly suppresses cellular motility, migration, and invasion in human osteosarcoma U2OS and HOS cells and represses the gene expression of C-C motif chemokine ligand 24 (CCL24) in U2OS cells [45]. Manipulation of CCL24 levels influenced the motility of U2OS and HOS cells as cell migration and invasion can be enhanced by the addition of recombinant human CCL24 and can be attenuated by the silencing of CCL24. While both inhibitors of JNK1/2 (SP600125 and DN-JNK) exaggerate melatonin’s attenuation of the expression of CCL24 mRNA and the migratory potential in U2OS cells, the inhibitor of ERK1/2 (U0126) cannot affect melatonin’s actions. Altogether, U2OS and HOS cell-derived CCL24 contributes to cellular invasion and migration through the upstream JNK signaling pathway; this finding implies a promotional role of CCL24 in osteosarcoma metastasis; the action is inhibited by melatonin.

### 4.3. Synergistic Effects and Utilization of Micro/Nanoparticles Delivery and Inclusion Complex Systems in Human Osteosarcoma

In managing patients diagnosed with any form of osteosarcoma, powerful therapeutic agents with less to no harmful side effects are the mainstay. Numerous approaches have been undertaken to improve survival rates and to diminish adverse side effects of drugs therapy, including the concomitant use of chemotherapy with melatonin due to its efficacy and safety [78,79]. Even in in vitro studies unresponsive to melatonin alone, the pineal hormone may advance the cytostatic and the cytotoxic effects triggered by other compounds or conventional drugs. Accumulating evidence suggests beneficial effects of the co-administration of melatonin with conventional drugs, which leads to synergistic effects, thus increasing cancer cell inhibition [48,50], indicating that melatonin is preeminently suitable as a drug in combination chemotherapy. For osteosarcoma, it is well perceived that melatonin inhibits activity, blocks the cell cycle at the G1-stage, and induces apoptosis in human osteosarcoma SaOS-2 cells [80] (Table 3). While melatonin has an antagonistic effect with a lower concentration of cis-platinum, it has a synergistic effect with methotrexate or a higher concentration of cis-platinum.

With current chemotherapeutic schedules for osteosarcoma, lung metastasis is almost impossible to cure [81]. Melatonin modulates anti-apoptotic processes in normal cells and activates bone cell proliferation and differentiation at 1 nM–1 μM concentrations [82], whereas it inhibits the proliferation of bone cancer cells to trigger pro-apoptotic signals at 4–10 mM concentrations [65]. The idea of encapsulating the drugs inside a micro/nanoparticle, fabricated from biodegradable polymers for drug delivery systems, to control drug release at desired sites, has become very intriguing [83]. Owing to the relatively short half-life (30–57 min) of melatonin in the blood [84], melatonin loaded poly(d,l-lactide-co-glycolide) (PLGA) nanoparticles and microparticles have been employed to fortify the inhibitory effect of human osteosarcoma MG-63 cell proliferation, providing an expectation about the usage of melatonin as an adjunct to the routine chemotherapy of osteosarcoma [83]. Moreover, 9 mM of melatonin concentration released from the melatonin/2-hydroxypropyl-β-cyclodextrin (HPβCD) inclusion complex loaded chitosan scaffolds causes time dependent cell death by reducing the proportion of the cells in the G2/M phase rather than the S phase, so the inclusion complex can be considered an alternative system for human osteosarcoma therapy [85].

## 5. Conclusions

Cellular and molecular signaling pathways, including oxidative stress, proliferation, apoptosis, and metastasis, involved in osteosarcoma, have been partially clarified in the past decade. Several in vitro and animal studies have evaluated the effect of melatonin on osteosarcoma, including synergistic effects with conventional chemotherapies and utilization of micro/nanoparticles delivery and inclusion complex systems for fortifying the effect. Accumulating evidence from experimental studies has supported the anti-osteosarcoma properties of melatonin, which may be a promising candidate as an adjuvant agent in osteosarcoma treatment. However, more studies are needed to completely elucidate the synergistic effect of melatonin with other compounds in osteosarcoma, both to raise success rates of therapy and also to abolish side effects. As melatonin is a potential therapeutic agent, we herein address several key concerns regarding its potential for treating patients with osteosarcoma. Finally, we need robust evidence from carefully designed and conducted clinical trials to underpin the treatment of osteosarcoma patients, particularly in young people.

## Figures and Tables

**Table 1:** Cytotoxic activity of melatonin in human osteosarcoma in vitro and in vivo.

Action	Pathway	Cell Line/In Vivo	Dose	Reference
No effect on the growth, morphology or cell cycle		MG-63	10^−5^–10^−13^ M	Panzer A, et al. 1998. [64]
Abundant expression of MT1-mRNA		HOS, MG-63		Toma CD, et al. 2007. [62]
Rescues cardiolipin-dependent mitochondrial dynamics-associated mitochondrial pathologies	Reduces mitochondrial ROS, cell death, and depletion of cardiolipin to improve retardation of mitochondrial movement and dynamics	143B	100 μM	Peng TI, et al. 2012. [63]
Inhibits proliferation	Down-regulates cyclin D1 and CDK4, (G1 phase), and cyclin B1 and CDK1 (G2/M phase)	MG-63	4(−10) mM	Liu L, et al. 2013. [65]
Induces apoptosis, increases ROS, and decreases GSH	Down-regulates SIRT1 and mitochondrial apoptotic pathway, and up-regulates acetylated-p53	SOSP-9607	(250−)1000 μM	Cheng Y, et al. 2013. [67]
Reduces adhesion and migration			12.5–50 μM	
Inhibits proliferation, induces G1 and G2/M phase arrest, and down-regulates cyclin D1, CDK4, cyclin B1 and CDK1	Inhibits the ERK1/2 pathway	MG-63	4 mM	Liu L, et al. 2016. [66]

MT1: melatonin receptor 1; ROS: reactive oxygen species; CDK: cyclin-dependent kinase; GSH: glutathione; SIRT1: sirtuin 1; ERK: extracellular signal-regulated protein kinase; and MG-63, HOS, 143B, and SOSP-9607: human osteosarcoma cell lines.

**Table 2:** Anti-metastatic effects of melatonin in human osteosarcoma in vitro and in vivo.

Action	Pathway	Cell Line/In Vivo	Dose	Reference
Induces apoptosis, increases ROS, and decreases GSH	Down-regulates SIRT1 and mitochondrial apoptotic pathway, and up-regulates acetylated-p53	SOSP-9607	(250−)1000 μM	Cheng Y, et al. 2013. [67]
Reduces adhesion and migration			12.5–50 μM	
Reduces the number (anti-proliferation)	Suppresses EMT via downregulation of SOX9, via the ERK pathway	Stem cell	>0.5 mM	Qu H, et al. 2018. [77]
Suppresses migration and invasion		U2OS, HOS	0.5 mM	
Inhibits initiation and metastasis in vivo		Mice model	100 mg/kg	
Inhibits motility, migration and invasiveness, and the CCL24 gene expression	Attenuates invasion and migration by suppression of CCL24 through inhibition of the JNK pathway	U2OS, HOS	2 mM	Lu KH, et al. 2018. [45]
Recombinant human CCL24 enhances migration and silencing of CCL24 attenuates migration and invasion				
Suppresses CCL24 and anti-metastasis through inhibition of the JNK pathway				

ROS: reactive oxygen species; GSH: glutathione; SIRT1: sirtuin 1; EMT: epithelial–mesenchymal transition; SOX9: a transcription factor; CCL24: C-C motif chemokine ligand 24; JNK: c-Jun N-terminal kinase; and SOSP-9607, U2OS, and HOS: human osteosarcoma cell lines; Stem: osteosarcoma stem cells.

**Table 3:** Synergistic effects and micro/nanoparticles delivery and inclusion complex systems of melatonin in human osteosarcoma in vitro and in vivo.

Action	Pathway	Cell Line/In Vivo	Dose	Reference
Inhibits cell activity, blocks the cell cycle at G1-stage, and induces apoptosis	Blocks the cell cycle at G1-stage	SaOS-2	0.5–5 mM	Wang YP, et al. 2015. [80]
An antagonistic effect with lower concentration of cis-platinum			1 mM	
A synergistic effect with methotrexate or higher concentration of cis-platinum				
Melatonin releasing PLGA micro/nanoparticles increases the inhibitory effect	Increases the inhibitory effect of proliferation	MG-63		Altındal DÇ, et al. 2016. [83]
Melatonin/HPβCD inclusion complex loaded into chitosan scaffolds causes cell death	Melatonin/HPβCD system reduces the proportion in the G2/M phase rather than S phase	MG-63	9 mM	Topal B, et al. 2015. [85]

PLGA: poly(d,l-lactide-co-glycolide); HPβCD: 2-hydroxypropyl-β-cyclodextrin; MG-63, SaOS-2: human osteosarcoma cell lines.

## Abbreviations

BAX	Bcl-2-associated X protein
Bcl-2	B-cell lymphoma/leukemia 2
CCL24	C-C motif chemokine ligand 24
EMT	epithelial–mesenchymal transition
ERK	extracellular signal-regulated protein kinase
GSH	glutathione
JNK	c-Jun N-terminal kinase
MT1	melatonin receptor 1
ROS	reactive oxygen species
SIRT1	sirtuin 1
